# Effect of Rapid Heating and Cooling Conditions on Microstructure Formation in Powder Bed Fusion of Al-Si Hypoeutectic Alloy: A Phase-Field Study

**DOI:** 10.3390/ma15176092

**Published:** 2022-09-02

**Authors:** Masayuki Okugawa, Yuya Furushiro, Yuichiro Koizumi

**Affiliations:** 1 Division of Materials and Manufacturing Science, Graduate School of Engineering, Osaka University, 2-1 Yamadaoka, Suita, Osaka 565-0871, Japan; 2 Anisotropic Design & Additive Manufacturing Research Center, Osaka University, 2-1 Yamadaoka, Suita, Osaka 565-0871, Japan

**Keywords:** additive manufacturing, Al-Si hypoeutectic alloy, multiphase-field simulation, rapid heating and cooling, grain refinement

## Abstract

Al alloy parts fabricated by powder bed fusion (PBF) have attracted much attention because of the degrees of freedom in both shapes and mechanical properties. We previously reported that the Si regions in Al-Si alloy that remain after the rapid remelting process in PBF act as intrinsic heterogeneous nucleation sites during the subsequent resolidification. This suggests that the Si particles are crucial for a novel grain refinement strategy. To provide guidelines for grain refinement, the effects of solidification, remelting, and resolidification conditions on microstructures were investigated by multiphase-field simulation. We revealed that the resolidification microstructure is determined by the size and number of Si regions in the initial solidification microstructures and by the threshold size for the nucleation site, depending on the remelting and resolidification conditions. Furthermore, the most refined microstructure with the average grain size of 4.8 µm is predicted to be formed under conditions with a large temperature gradient of *G*_sol_ = 10^6^ K/m in the initial solidification, a high heating rate of *HR* = 10^5^ K/s in the remelting process, and a fast solidification rate of *R*_resol_ = 10^−1^ m/s in the resolidification process. Each of these conditions is necessary to be considered to control the microstructures of Al-Si alloys fabricated via PBF.

## 1. Introduction

Additive manufacturing (AM) has garnered substantial attention owing to its capability to fabricate parts with complex shapes as well as control internal microstructures through variations in process parameters [1]. With regard to metal AM, powder bed fusion (PBF), wherein the metal powder is repeatedly melted and solidified using either laser or electron beam irradiation, has become mainstream. Furthermore, Al alloy parts fabricated by PBF-based AM tend to have superior characteristics, such as high specific strength, high fatigue resistance, and a high degree of physical flexibility. Therefore, they have been widely adopted in industrial fields, such as manufacturing automobiles, motorcycles, airplanes, and spacecraft [2,3,4,5,6,7,8,9,10,11,12,13,14,15,16,17,18]. Al alloys with fine equiaxed grain microstructures exhibit excellent characteristics with respect to the aforesaid applications; therefore, inoculants are introduced as heterogeneous nucleation sites for further microstructure refinement [5,6,7,8,9,19,20,21]. However, the cost of the inoculants, which are rare earth elements, leads to a considerable increase in the price of the powder. Consequently, there is demand for a new grain refinement technique in PBF-type AM.

Generally, the solidification microstructures in metals can be determined by the solidification conditions, such as the temperature gradient, *G,* at the liquid/solid interface and the solidification rate (i.e., the velocity of the solid/liquid interface), *R*, based on Hunt’s columnar–equiaxed transition (CET) criteria [22,23,24,25,26,27,28,29]. According to the CET criteria, because the melt-pool boundary where the solidification begins has a large *G* and a small *R*, it can be predicted with a high degree of confidence that columnar crystals form more easily in its vicinity, whereas equiaxed grains form more easily at the center of the melt pool. On the contrary, in the case of Al-Si alloys, equiaxed crystals are often formed in the vicinity of the melt-pool boundaries, whereas columnar crystals are formed near the center of the melt pool, thus contradicting the predictions based on the CET criteria [2,10]. The elucidation of the mechanism that underlies this phenomenon is a crucial challenge that would allow for the fabrication of metals with improved material properties through better control over the microstructures in AM. Through multiphase-field (MPF) simulations, we have demonstrated that the Si particles in eutectic regions near the melt-pool boundary of a solidified Al-Si alloy remain, even after the remelting process, because of the rapid heating rate of PBF-type AM; furthermore, such remaining crystalline Si particles act as heterogeneous nucleation sites for α-Al equiaxed crystalline grains [30]. This process has been proposed as the grain refinement and formation mechanism near the melt-pool boundaries.

**Figure 1:**
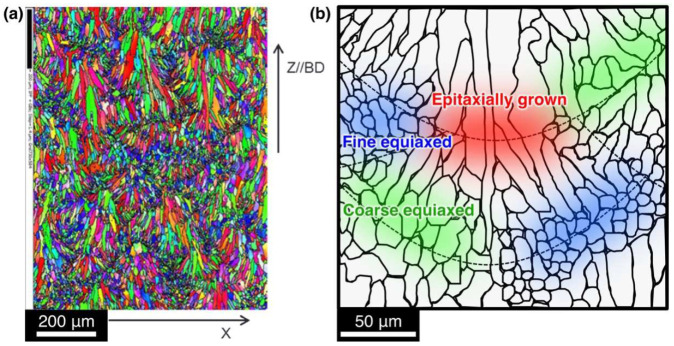
(**a**) SEM-EBSD inverse-pole-figure (IPF) orientation maps of AMed AlSi10Mg. Adapted from [2], copyright (2017), with permission from Elsevier. (**b**) Schematic illustration of the inhomogeneous microstructure near the melt-pool boundary. The dashed lines indicate the fusion lines.

In the Al-Si alloy parts fabricated by PBF-type AM [2,10], the microstructures near the melt-pool boundaries vary by region. As shown in Figure 1, equiaxed crystalline grains and epitaxially grown columnar crystals are observed. In the PBF AM process, the defined solidification conditions can vary within a single melt-pool [29]. Thus, different microstructures can form under diverse solidification, remelting, and resolidification conditions because of the varied overlapping of melt-pools. Conversely, this suggests that a finer grain microstructure can be obtained by controlling the scan pitch and layer thickness. In this context, this study focuses on the microstructures observed in Al-Si alloy parts manufactured by PBF-based AM to develop a new grain refinement technique for Al alloy AM. Specifically, in this study, we have investigated the effects of solidification, remelting, and resolidification conditions on the final resolidified microstructure of MPF simulations to provide guidelines for grain refinement in the PBF-type AM of Al-Si hypoeutectic alloys.

## 2. Methods

Two-dimensional MPF simulations of an Al-10 mass% Si model were performed using the Microstructure Evolution Simulation Software (MICRESS) [31,32] with TQ-Interface for Thermo-Calc [33]. In the MPF method, the distribution of phase-field variables, which indicates the probability of the existence of each phase, and the distribution of solute concentration were manipulated based on their time evolution equation, and structural change was simulated based on the changes in the aforenoted distributions. 

The liquid phase, α-Al (FCC structure) phase, and Si (diamond structure) phase were assumed as the constituent phases. The Gibbs free energy and the diffusion potential of the Al-Si binary system were calculated using CALPHAD data [34]. Heterogeneous nucleation was assumed for the solid Si-phase and for α-Al solids based on the α-Al solid/liquid and solid Si-phase/liquid interfaces, respectively. In accordance with the reports on MPF simulations of the solidification of Al-Si alloys by Eiken et al. [35,36], the interface energies corresponding to α-Al/liquid, Si/liquid, α-Al/α-Al, and α-Al/Si were set as 165, 352, 150, and 380 mJ m^−2^, respectively. A value of 5.0 × 10^−10^ m^4^ J^−1^ s^−1^ was used for the interface mobility for the boundary between the solid and liquid phases, following Nomoto et al. [37]. The simulation domain size was 50 × 100 μm. The grid size, Δ*x*, and interface width were set as 1 µm and 3.5 µm, respectively. As an initial condition, five crystalline Al nuclei with random orientations were placed at the bottom of the simulation domain, and the remaining portion was set as an Al-10 mass% Si liquid. The temperature at the bottom was initially set at 865 K, i.e., 20 K below the liquidus temperature. 

This model was solidified, remelted, and resolidified under conditions ①–⑤ shown in Table 1. First, solidification was simulated under the condition of a temperature gradient, *G*_sol_, and an interface velocity, *R*_sol_. Then, the solidified model was remelted at *HR*. After the melting of the material from the upper edge by up to 75 μm, i.e., 75% of the simulation domain, the remelted model was resolidified under the condition of a temperature gradient, *G*_resol_, and an interface velocity, *R*_resol_. The Si concentration and the crystal orientation distributions of the microstructures formed after each process were compared.

## 3. Results and Discussion

**Figure 2:**
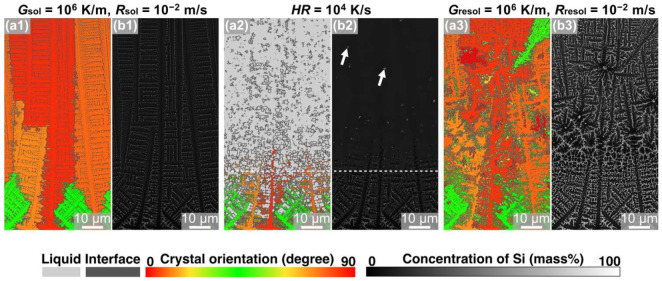
Snapshots of MPF simulation of solidification—remelting—resolidification process of Al-10 mass% Si alloys under condition ①: (**a1**,**b1**) solidified with *G*_sol_ = 10^6^ K/m and *R*_sol_ = 10^−2^ m/s, (**a2**,**b2**) remelted with *HR* = 10^4^ K/s, and (**a3**,**b3**) resolidified with *G*_resol_ = 10^6^ K/m and *R*_resol_ = 10^−2^ m/s. The color indicates the distribution of (**a**) crystal orientation and (**b**) the concentration of Si. The white dashed line in (**a2**,**b2**) indicates the fusion line, and the arrows in (**b2**) indicate the remaining Si-phase particles.

Figure 2 shows MPF models of solidified (Figure 2(a1,b1)), remelted (Figure 2(a,b2)), and resolidified (Figure 2(a3,b3)) Al-Si hypoeutectic alloys under condition ①, colored based on the crystal-orientation angle (Figure 2(a1–a3)) and Si concentration (Figure 2(b1–b3)). As shown in Figure 2(a1), columnar α-Al crystals with a width of approximately 5 μm appeared elongated along the temperature gradient direction under the solidification condition defined by *G*_sol_ = 10^6^ K/m and *R*_sol_ = 10^−2^ m/s. Crystal grains in the [100] orientation grew preferentially, and the [100]-oriented crystals dominated in the solidified model. Eutectic regions comprising the α-Al and Si phases formed between dendritic columnar crystals and between secondary arms. The solidified Al-Si alloy model was heated at *HR* of 10^4^ K/s until the melting of 75 μm from the upper edge, as shown in Figure 2(a2,b2). The white dashed line in Figure 2(a2) indicates the fusion line (defined as the isotemperature line for the liquidus of the Al-10 mass% Si alloy). The solidified microstructure melted inhomogeneously: columnar dendrites of the α-Al phase were fully melted, whereas the regions with the eutectic microstructure were melted partially, and the crystalline solid Si-phase particles remained, as indicated by the arrows in the Si concentration map (Figure 2(b2)). The remelted Al-Si eutectic alloy model cooled again and resolidified, as shown in Figure 2(a3,b3). Dendrites grew isotropically near the fusion line, and equiaxed grains approximately 21.9 μm in diameter were formed. The center positions of the equiaxed grains were the same as those of the remaining Si particles observed in the remelted model in Figure 2(a2,b2). This indicates that the remaining Si particles act as heterogeneous nucleation sites during the resolidification process under high heating and cooling rates such as those in the PBF-type AM process. 

**Figure 3:**
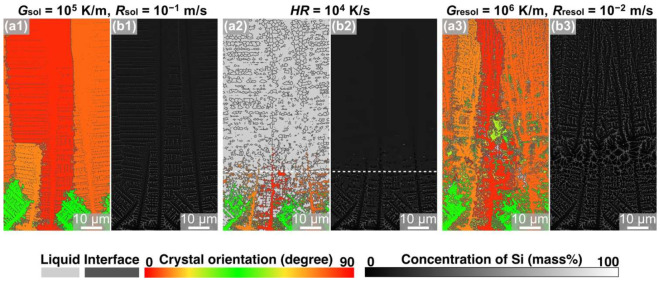
Snapshots of MPF simulation of solidification—remelting—resolidification process in Al-10 mass% Si alloys under condition ②: (**a1**,**b1**) solidified with *G*_sol_ = 10^5^ K/m and *R*_sol_ = 10^−1^ m/s, (**a2**,**b2**) remelted with *HR* = 10^4^ K/s, and (**a3**,**b3**) resolidified with *G*_resol_ = 10^6^ K/m and *R*_resol_ = 10^−2^ m/s. The color indicates (**a**) the crystal orientation and (**b**) the concentration of Si.

MPF simulations of the solidification, remelting, and resolidification processes were performed under condition ②, in which only the initial solidification condition is changed from that of condition ①: *G*_sol_ = 10^5^ K/m and *R*_sol_ = 10^−1^ m/s. Figure 3 shows the MPF models, colored based on the crystal-orientation angle (Figure 3(a1–a3)) and Si concentration (Figure 3(b1–b3)), after each process under condition ②. When the alloy model was cooled from the liquid phase at a constant temperature gradient (Figure 3(a1)), the columnar crystals were formed and crystal grains in the [100] orientation dominated. Regions with high concentrations of Si can be observed between the dendrites, indicating that eutectic structures were formed between the dendrites. Note that the microstructures formed between the dendrites were finer than those formed under condition ①. This solidification model was then heated at *HR* = 10^4^ K/s, and the upper portion within 75 µm of the top of the simulation domain was remelted (Figure 3(a2)). Contrary to the case under condition ①, crystalline Si particles did not remain in the remelted model (Figure 3(b2)). There were no remaining crystalline Si particles because the sizes of the Si phases in the eutectic regions in the solidification model were sufficiently small that the Si phases completely melted during the rapid heating process. The remelted model was resolidified under the resolidification conditions of *G*_resol_ = 10^6^ K/m and *R*_resol_ = 10^−2^ m/s (Figure 2(a3)). Columnar crystals grew epitaxially from the unmelted crystal placed outside. Furthermore, a previous study demonstrated the formation of equiaxed crystalline grains near the melt-pool boundary in Al-Si hypoeutectic alloy using Si crystalline particles as heterogeneous nucleation sites [30]. Under condition ②, Si crystalline particles did not remain after the remelting process, and therefore, equiaxed crystalline grains were absent from the resolidification microstructure.

**Figure 4:**
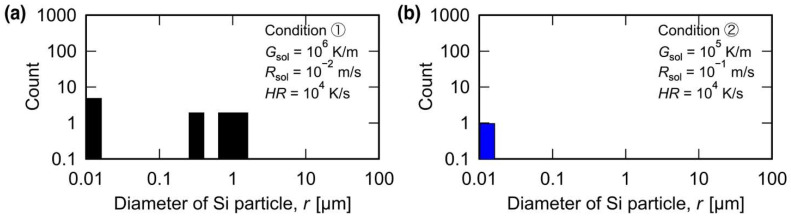
Histogram of the size and number of Si particles after melting under (**a**) condition ① where *G*_sol_ is 10^6^ K/m and *R*_sol_ is 10^−2^ m/s during solidification and *HR* is 10^4^ K/s during melting and (**b**) condition ② where *G*_sol_ is 10^5^ K/m and *R_sol_* is 10^−1^ m/s during solidification and *HR* is 10^4^ K/s during melting.

When the solidification conditions were set to *G*_sol_ = 10^6^ K/m and *R*_sol_ = 10^−2^ m/s, equiaxed crystalline grains were formed during the resolidification process, wherein the Si crystalline particles served as the nucleation sites (Figure 2(a3)). On the other hand, when the solidification conditions were set to *G*_sol_ = 10^5^ K/m and *R*_sol_ = 10^−1^ m/s, no remaining Si crystalline particles were found after the remelting process, and columnar crystals were formed after the resolidification process (Figure 3(a3)). The differences in the remelting behavior of Si phases in the eutectic regions of the solidified microstructures can be attributed to the difference in the sizes of the Si particles in the initial solidification microstructure. Therefore, the size and number of Si particles in the initial solidification microstructures under conditions ① and ② were measured. The average size of the Si particles in the model solidified under condition ① at *G*_sol_ = 10^6^ K/m and *R*_sol_ = 10^−2^ m/s was 230 nm with a total of 2175 particles, whereas the average size of the Si particles in the model solidified under solidification condition ② at *G*_sol_ = 10^5^ K/m and *R*_sol_ = 10^−1^ m/s was 170 nm with a total of 2572 particles. The different solidification conditions affected both the average size and amount of Si particles. Figure 4 shows the size distributions of the remaining Si crystalline particles after the remelting process under conditions ① and ②. Coarser Si particles were formed after the solidification process under a lower temperature gradient, *G*_sol_, even at the same cooling rate. 

Multiple Si particles remained under condition ① (Figure 4a), whereas only a single Si particle remained under condition ② (Figure 4b). These results suggest that the initial solidification condition determines the Si particle size and the amount in the initial solidification microstructure. Consequently, the Si particle size and amount determine the number of remaining Si crystalline particles, which act as the heterogeneous nucleation sites for equiaxed crystalline grains. Moreover, although Si crystalline particles approximately 1 μm in diameter remained under condition ②, an equiaxed crystalline grain did not appear during the resolidification process. This result indicates that Si crystalline particles with critical sizes exceeding 1 μm are essential for heterogeneous nucleation under the resolidification condition of *G*_resol_ = 10^6^ K/m and *R*_resol_ = 10^−1^ m/s. 

**Figure 5:**
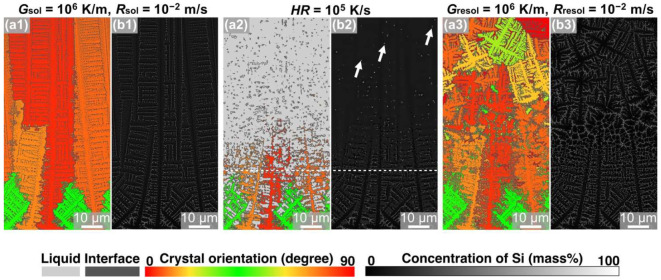
Snapshots of MPF simulation of solidification—remelting—resolidification process in Al-10 mass% Si alloys under condition ③: (**a1**,**b1**) solidified with *G*_sol_ = 10^6^ K/m and *R*_sol_ = 10^−2^ m/s, (**a2**,**b2**) remelted with *HR* = 10^5^ K/s, and (**a3**,**b3**) resolidified with *G*_resol_ = 10^6^ K/m and *R*_resol_ = 10^−2^ m/s. The color indicates (**a**) the crystal orientation and (**b**) the concentration of Si.

**Figure 6:**
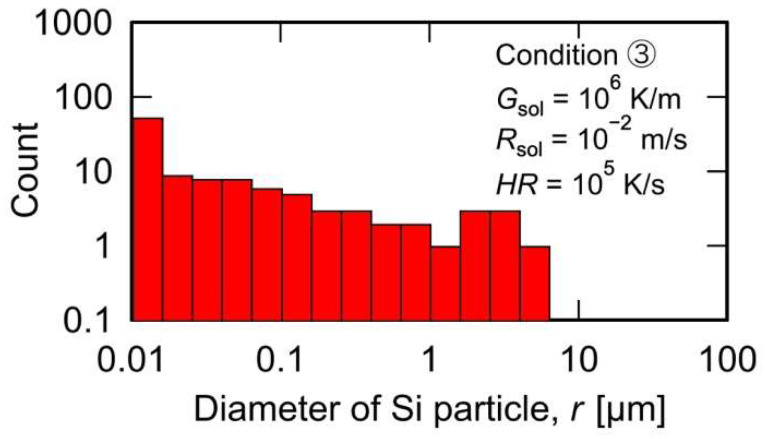
Histogram of the size and number of Si particles after melting under condition ③, where *G*_sol_ is 10^6^ K/m and *R*_sol_ is 10^−2^ m/s during solidification and *HR* is 10^5^ K/s during melting.

MPF simulations of the solidification, remelting, and resolidification processes were performed under condition ③ with *HR* set at 10^5^ K/s to reveal the effect of *HR* during the remelting process on the microstructure formation. Figure 5 presents the crystal orientation map (Figure 5(a1–a3)) and the Si concentration distribution map (Figure 5(b1–b3)) obtained after each process. The solidification model with a microstructure comprising columnar crystals (Figure 5(a1)) was heated at *HR* = 10^5^ K/s, and the upper region of the simulation domain was remelted to 75 µm (Figure 5(a2)). In the Si concentration map of the melted model (Figure 5(b2)), regions with high Si concentrations, i.e., the remaining Si crystalline particles, were formed within the liquid phase. The number of remaining Si crystalline particles was higher than that in the remelted model under condition ① (*HR* = 10^4^ K/s; Figure 2(a2,b2)). When the remelted model was resolidified under *G*_resol_ = 10^6^ K/m and *R*_resol_ = 10^−2^ m/s, equiaxed crystalline grains with a diameter of approximately 12.3 µm were formed in the upper portion of the simulation domain (Figure 5(a3,b3)). The model resolidified under condition ③ (*HR* = 10^5^ K/s) exhibited finer equiaxed crystalline grains than that under condition ① (*HR* = 10^4^ K/s). As mentioned above, these equiaxed crystalline grains were formed using the Si crystalline particles as heterogeneous nucleation sites. Thus, the Si particle size distribution in the model remelted under condition ③ was measured, as shown in Figure 6. Compared to the model remelted under condition ① (*HR* = 10^4^ K/s, Figure 4a), the model remelted under condition ③ (*HR* = 10^5^ K/s, Figure 6) featured more coarse Si particles with sizes exceeding 1 μm; hence, this is suggested to be the critical size for heterogeneous nucleation sites under the resolidification conditions of *G*_resol_ = 10^6^ K/m and *R*_resol_ = 10^−2^ m/s. Furthermore, it was also concluded that the number of Si crystalline particles exceeding this critical size (and thus serving as nucleation sites) was higher under condition ③ than under condition ①, which enabled finer solidification microstructure formation in the former condition.

**Figure 7:**
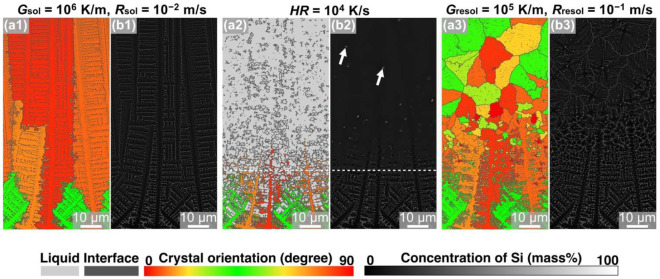
Snapshots of MPF simulation of solidification—remelting—resolidification process in Al-10 mass% Si alloys under condition ④: (**a1**,**b1**) solidified with *G*_sol_ = 10^6^ K/m and *R*_sol_ = 10^−2^ m/s, (**a2**,**b2**) remelted with *HR* = 10^4^ K/s, and (**a3**,**b3**) resolidified with *G*_resol_ = 10^5^ K/m and *R*_resol_ = 10^−1^ m/s. The color indicates (**a**) the crystal orientation and (**b**) the concentration of Si.

To examine the effect of the resolidification rate on structure formation, simulations involving the Al-Si alloy model were performed under condition ④. The crystal orientation maps (Figure 7(a1–a3)) and the Si concentration distribution maps (Figure 7(b1–b3)) obtained after the process are shown in Figure 7. The solidified model has a columnar crystal microstructure (Figure 7(a2)), and the remelted model contains the remaining Si crystalline particles (Figure 7(a1)). For the remelted model resolidified under the conditions of *G*_resol_ = 10^6^ K/m and *R*_resol_ = 10^−1^ m/s, equiaxed crystals with a diameter of approximately 9.5 µm were observed (Figure 7(a3)). The model resolidified under condition ④ (*R*_resol_ = 10^−1^ m/s) featured finer equiaxed crystalline grains compared to the model resolidified under condition ① (*R*_resol_ = 10^−2^ m/s). As the solidification and remelting conditions were identical in both these cases, the difference in the microstructure formation was attributed to the resolidification conditions alone. Thereafter, the size of the Si crystalline particles that functioned as heterogeneous nucleation sites for the equiaxed crystals was measured. The critical sizes of the Si crystalline particles for heterogeneous nucleation under condition ① and condition ④ were 1.2 μm and 0.4 μm, respectively.

**Figure 8:**
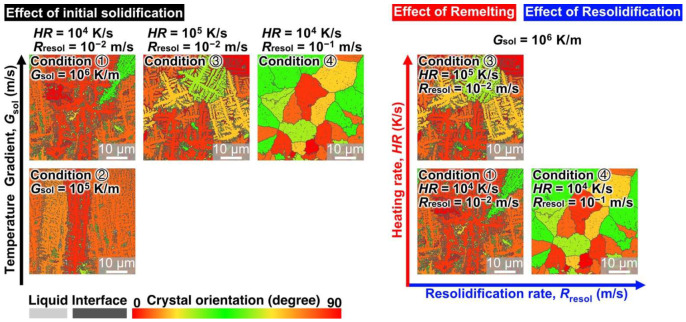
Summary of the microstructures of Al-10 mass% Si alloys predicted by MPF simulations under various solidification, remelting, and resolidification conditions.

**Figure 9:**
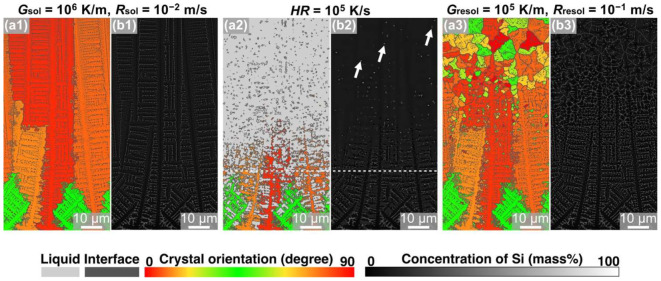
Snapshots of MPF simulation of solidification—remelting—resolidification process in Al-10 mass% Si alloys under condition ⑤: (**a1**,**b1**) solidified with *G*_sol_ = 10^6^ K/m and *R*_sol_ = 10^−2^ m/s, (**a2**,**b2**) remelted with *HR* = 10^5^ K/s, and (**a3**,**b3**) resolidified with *G*_resol_ = 10^5^ K/m and *R*_resol_ = 10^−1^ m/s. The color indicates (**a**) the crystal orientation and (**b**) the concentration of Si.

Figure 8 shows the MPF-predicted microstructures summarized by the solidification, remelting, and resolidification conditions. Based on the simulations, we found that, on increasing the number of Si particles exceeding a certain size after the initial solidification and rapid heating/cooling, the remelting and resolidification processes contributed to the formation of finer equiaxed grains in the Al-Si alloy during the AM process. Therefore, a microstructure with the finest grains is expected to be formed under a large *G*_sol_, high *HR*, and high *R*_resol_. Figure 9 shows the microstructure formed after the solidification, remelting, and resolidification processes under condition ⑤: solidification conditions of *G*_sol_ = 10^6^ K/m, *R*_sol_ = 10^−2^ m/s, and *HR* = 10^5^ K/s and resolidification conditions of *G*_sol_ = 10^5^ K/m and *R*_sol_ = 10^−1^ m/s. The model thus obtained featured the finest equiaxed crystal structures, approximately 4.8 µm in size (Figure 9(a3,b3)). 

**Figure 10:**
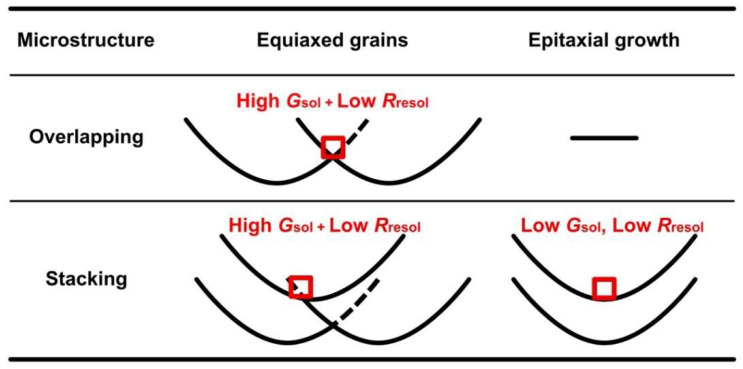
Microstructure formations near the melt-pool boundary of Al-10 mass% Si alloys based on the results of MPF simulations and the distribution of *G* and *R* in a melt pool [29].

The temperature gradient, *G*, is the highest in the regions near the fusion line, whereas the interface velocity, *R,* is the highest at the center of the melt region in an actual AM process [29]. Part of the variation in experimental microstructure formation near the melt-pool boundary of Al-10 mass% Si alloys (Figure 1) can be explained based on the distribution of *G* and *R* in a melt pool and the results of MPF simulations (Figure 8), as shown in Figure 10. Remelt and resolidification occur in overlapped regions of melt pools and in a stacking process of upper layers, and initial solidified microstructures are different, region by region. Equiaxed grains are predicted to be formed by the remelting and resolidification of the edge of the melt pool due to high *G*_sol_ and low *R*_resol_ conditions, whereas epitaxial growth is predicted to occur from the remelting and resolidification of the center of the melt pool due to low *G*_sol_ and low *R*_resol_ conditions. However, to fully understand the microstructure formation, including the variety in the size of equiaxed grains, considerations of the distribution of melting conditions in a melt pool of Al-10 mass% Si are required. Hence, an analysis of melting and solidifying behavior is underway with the combined methods of laser-irradiation experiments and computational thermal fluid dynamics simulation. 

In such an analysis, the various material properties, such as laser absorptivity [38], heat conductivity [39], surface tension [40], and evaporation pressures, are important since they strongly affect the melting and subsequent solidification behaviors and their spatial distribution.

## 4. Conclusions

In this work, we investigated the effects of the solidification, remelting, and resolidification conditions on the microstructures of Al-Si hypoeutectic alloys via multiphase-field simulations. 

Simulations conducted using different initial solidification conditions revealed that the size and number of Si particles in the initial solidification microstructures affect the resolidified microstructures. Under a high temperature gradient, *G*_sol_, coarse Si particles were formed in the initial solidified structure. These coarse Si particles remained, even after the remelting process, and served as heterogeneous nucleation sites for equiaxed grains during the resolidification process. By contrast, under low *G*_sol_, no coarse Si particles were formed after the initial solidification, and columnar crystals grew epitaxially during the resolidification process. 

The heating rate, *HR*, during the remelting process and the solidification rate, *R*_resol,_ during resolidification influenced the size of the equiaxed crystalline grains: finer equiaxed crystalline grains were formed under high *HR* and *R*_resol_. It is suggested that the number of Si particles acting as heterogeneous nucleation sites increased because the time required for the remelting and resolidification processes was shorter under conditions involving high *HR* and *R*_resol_.

Furthermore, it is necessary to consider the conditions of each of the solidification, remelting, and resolidification processes to control the microstructures of Al-Si alloys fabricated via PBF-type AM. Based on the results of this study, microstructures with significantly finer grains can likely be formed under conditions involving large *G*_sol_, high *HR*, and high *R*_resol_, and such conditions can be achieved by controlling the beam power, beam scanning speed, and scan pitch. 

## Figures and Tables

**Table 1:** Solidification, remelting, and resolidification conditions used in the MPF simulations.

Condition	Solidification		Remelting	Resolidification	
	*G*_sol_ (K/m)	*R*_sol_ (m/s)	*HR* (K/s)	*G*_resol_ (K/m)	*R*_resol_ (m/s)
①	10^6^	10^−2^	10^4^	10^6^	10^−2^
②	10^5^	10^−1^	10^4^	10^6^	10^−2^
③	10^6^	10^−2^	10^5^	10^6^	10^−2^
④	10^6^	10^−2^	10^4^	10^5^	10^−1^
⑤	10^6^	10^−2^	10^5^	10^5^	10^−1^

## Data Availability

Not applicable.
